# Dolphin semen metabolome characterization and relationships with sperm quality

**DOI:** 10.1007/s11306-026-02552-z

**Published:** 2026-11-13

**Authors:** Jacquline Rich, Hussain Abdulla, Gisele A. Montano, Dara N. Orbach

**Affiliations:** 1 Department of Life Sciences, Texas A&M University – Corpus Christi, Corpus Christi, TX USA; 2 Department of Physical and Environmental Sciences, Texas A&M University – Corpus Christi, Corpus Christi, TX USA; 3 SeaWorld Busch Gardens Species Preservation Laboratory, United Parks and Resorts, SeaWorld California, San Diego, CA USA; 4 United Parks and Resorts, Corporate Zoological Operations, SeaWorld Orlando, Orlando, FL USA

**Keywords:** Cetacean, Mass spectrometry, Metabolomics, Pathway analysis, Semen quality

## Abstract

**Introduction:**

Identification and quantification of metabolites in semen can augment assisted reproductive technologies by indicating reproductive health and fertility. Species-specific semen metabolites associated with improved semen quality remain largely unknown in exotic species due to limited sample availability.

**Objectives:**

Study objectives were to establish metabolome profiles of common bottlenose dolphin (*Tursiops truncatus*) semen and seminal plasma and identify metabolites associated with semen quality.

**Methods:**

Semen was collected from sexually mature common bottlenose dolphins (aged 17–46 years) trained to perform voluntary semen collection behavior. One to five semen samples were collected from each of four dolphins in managed care, resulting in 12 ejaculates for metabolomic analyses. Semen quality parameters were measured including volume, pH, and sperm concentration, motility, kinematics, and viability. Metabolites in whole semen and sperm-free seminal plasma were analyzed using ultra-high performance liquid chromatography coupled with Orbitrap Fusion Tribrid mass spectrometry. Metabolome profile variability was assessed using principal component analyses with permutational multivariate analysis of variance and volcano plots with t-tests. Metabolomic pathway analyses were conducted using a species-specific systems biology markup language (SBML) metabolite model. Correlations between semen quality parameters and normalized metabolite peak areas were assessed using repeated-measures correlation.

**Results:**

An untargeted analysis identified 2,198 metabolites in dolphin semen and seminal plasma. Biological matrix, individual variation, and collection date contributed to differences in metabolome profiles. The relative abundances of 353 metabolites differed significantly between semen and seminal plasma (*p* < 0.05). Targeted analysis detected eight hormones in semen and/or seminal plasma. Pathway enrichment analysis indicated inter-individual variation including enriched nucleotide metabolism, protein degradation, and transport reaction pathways. Two individuals exhibited enriched inflammatory, reactive oxygen species detoxification, and antioxidant metabolism pathways, suggesting oxidative stress. A significant correlation (*p* < 0.0005) occurred between 98 distinct metabolites and semen quality parameters.

**Conclusion:**

This study provides the first semen metabolome profile for any marine mammal species and identifies novel metabolomic biomarkers of dolphin semen quality.

**Supplementary Information:**

The online version contains supplementary material available at https://doi.org/10.1007/s11306-026-02552-z.

## Introduction

As infertility rates rise globally across taxa, there is an urgent need to improve understanding of the mechanisms controlling infertility (Eisenberg et al., 2023; Brander et al., 2026). Declining semen quality may largely contribute to male factor infertility in human and animal models (Agarwal et al., 2015; Levine et al., 2017; Brander et al., 2026). Infertility among individuals with clinically high-quality semen metrics indicate that sperm motility, concentration, and morphology are insufficient parameters to assess male reproductive health alone (Eisenberg et al., 2023; Hassanine et al., 2026). Advanced instrumentation has enabled multi-omics analyses of semen and seminal plasma of humans and terrestrial mammals that have identified molecular mechanisms modulating male fertility potential (Wagner et al., 2023); however, multi-omics analyses of marine megafauna semen have not been conducted.

Over the past two decades, metabolomics has emerged as a powerful tool in infertility investigations (Mehrparavar et al., 2019). Sperm and seminal plasma contain abundant metabolites, or endogenous molecules generally < 1,500 dalton (Da), that have distinct roles in regulating semen quality (Schjenken & Robertson, 2014; Moreno-Ulloa, 2026). For example, hormones coordinate spermatogenesis and sperm behavior (Kumar & Singh, 2020; Osadchuk et al., 2023) while amino acids promote sperm motility by functioning as an energy source (Juyena & Stelletta, 2012). Metabolomic analyses demonstrate that semen quality parameters are influenced by semen molecular composition and interactions between semen metabolites, sperm cells, and the extracellular environment (Holt & Van Look, 2004).

As semen biochemistries are indicative of quality, specific metabolites can serve as biomarkers of reproductive health (Deng et al., 2020; Guvvala et al., 2020). Identification of metabolites associated with improved fertility requires species-specific investigations as metabolites can have differential functions that are interspecific. For example, alkaline phosphatase, an enzyme that functions to catalyze the hydrolysis of phosphates, possesses varied correlations with semen quality parameters between species (Alyethodi et al., 2022). Alkaline phosphatase activity was positively correlated with sperm concentration in swine (*Sus scrofa*; Rodríguez et al., 2013) while no correlation was found in closely related cattle (*Bos taurus indicus*; Alyethodi et al., 2022). Intraspecific differential enrichment of metabolic pathways among individuals can affect the semen metabolome and quality (Lu et al., 2026). Semen metabolome variability within and between individuals can be affected by ejaculatory frequency (Carlsen et al., 2004; Frangež et al., 2005), genetics (Carvalho et al., 2023; Hodge et al., 2023), season, and environmental parameters (Centola & Eberly, 1999; Jurewicz et al., 2009; Surisetti et al., 2024), among other factors.

The complex biochemical mechanisms regulating male fertility potential necessitate a species-specific approach to fertility studies. Systems Biology Markup Language (SBML) models store and share computational models of biological processes in an XML-based standardized language (Hucka et al., 2015). Systems biology approaches have been increasingly adopted to investigate species-specific metabolomic pathways associated with fertility potential (Razavi et al., 2017; Krog et al., 2022). Reproducible SBML models accessible by multiple softwares have identified novel pathways in human sperm metabolism associated with reduced fertility phenotypes (Asghari et al., 2017). SBML models for semen metabolomics analysis in animals have not been made publicly accessible.

Cetaceans (whales, dolphins, and porpoises) face increasing anthropogenic threats including habitat degradation, pollution, fisheries interactions, and climate change, resulting in 26% of cetacean species assessed by the International Union for Conservation of Nature at risk of extinction (International Union for Conservation of Nature SSC Cetacean Specialist Group, 2025). *Ex situ* conservation efforts including development of species-specific assisted reproductive technologies (ARTs) have supported *in situ* cetacean conservation (O’Brien & Robeck, 2010). Zoological institutions and aquaria often manage relatively small and genetically limited populations, where maintaining genetic diversity and minimizing inbreeding are major challenges (Ballou et al., 2010). Improved understanding of semen quality and reproductive physiology is essential to facilitate the exchange of genetic material among institutions, optimize breeding programs, and support sustainable population management (Martin et al., 2026).Despite established cetacean breeding programs and germplasm banks (Robeck & O’Brien, 2004; O’Brien & Robeck, 2010), metabolomic markers of semen quality have not been assessed in the semen of any cetacean. Identification of metabolomic profiles associated with improved semen quality could inform semen sample selection to augment existing cetacean ARTs. We used common bottlenose dolphins (*Tursiops truncatus*) due to the availability of males trained for voluntary semen collection and their history as a model species for development of cetacean ARTs (O’Brien & Robeck, 2010). As hormones in semen and seminal plasma affect semen quality in mammals (Juyena & Stelletta, 2012), a targeted hormone analysis was conducted alongside untargeted metabolome profiling. The purposes of this study were to: (1) establish metabolomic profiles of common bottlenose dolphin semen and sperm-free seminal plasma and (2) identify metabolites and metabolomic pathway enrichment associated with semen quality (e.g., sperm concentration, motility, and kinematic parameters). Metabolomic profiles and pathway enrichment were hypothesized to vary between semen and seminal plasma within and between individuals exhibiting ejaculates with variable semen quality.

## Methods

### Semen collection

Semen was collected from four sexually mature common bottlenose dolphins in managed care previously trained in voluntary semen collection using manual stimulation (Table 1; Keller, 1986). No adverse behavioral responses were observed in response to semen collection. All dolphins were in good health and were not administered any medications at the time of semen collection.

**Table 1:** Life history metadata for common bottlenose dolphins (*Tursiops truncatus*) included in the study.

Individual	Age (years)	Body Weight (lb)	Total offspring sired
Male 1	27	525	0
Male 2	46	685	2
Male 3	20	505	3
Male 4	17	470	0

**Fig. 1:**
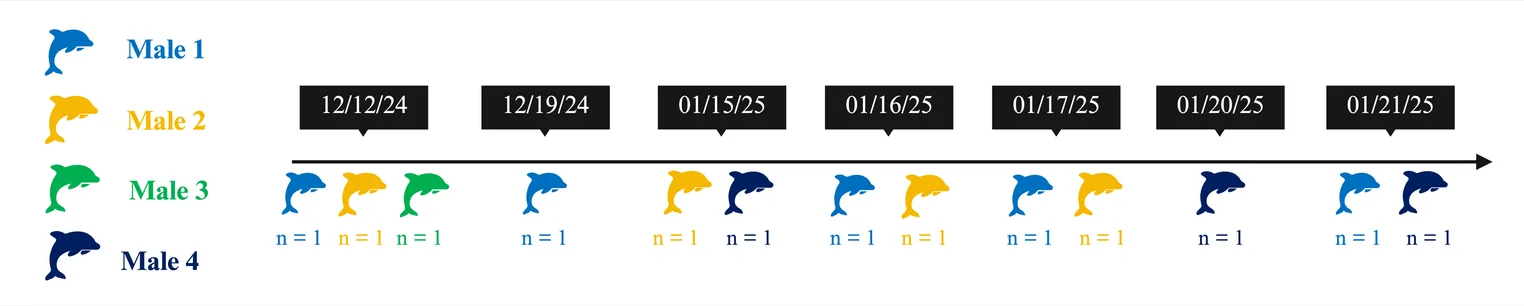
Timeline of semen collection from male common bottlenose dolphins (*Tursiops truncatus*). The colored icons represent individual males. The number below the icons denote the number of semen samples collected per male per date. Created with BioRender.com.

The dolphins were housed in a social group consisting exclusively of 14 males in an outdoor enclosure (28º 24’ 51” N) exposed to a natural photoperiod of approximately 10–11 daylight hours. The recirculated enclosure water was created by adding artificial saltwater to city tap water and maintained temperature between 16.5 and 23 °C and salinity between 25 and 26 parts per thousand. The dolphins are fully aquatic thus ambient air temperature was not relevant. The dolphins’ diet consisted of wild-caught capelin (*Mallotus villosus*) and herring (*Clupea harengus*), Mazuri^®^ Marine Mammal Supplements (Mazuri Exotic Animal Nutrition, St. Louis, MO), and isotonic water and gelatin to supplement hydration. Dolphins were trained to invert their bodies and hold their ventrum above the water’s surface where trainers induced erection using tactile stimulation. The penis tip was rinsed with a buffered solution upon full erection (225–245 mOsm and pH 7.3–7.4; O’Brien & Robeck, 2006). The penis tip was directed into a sterile plastic bag for semen collection. A total of 13 semen samples were collected over a six-week period with one to three semen samples collected from each male (Fig. 1). Semen samples were collected around the same time daily with a maximum of one sample collected per dolphin per day. As an osmolality assessment indicated probable saltwater contamination in one sample from Male 4 (osmolality > 350 mOsm/kg; Montano et al., 2012), this sample was excluded from analyses. No other samples were contaminated with urine or saltwater.

### Semen quality analysis

Semen processing and quality analyses occurred on-site immediately after collection. An aliquot of 1.5–10 mL semen from each sample was transferred to polystyrene tubes (BD Falcon™, Becton Dickinson, Franklin Lakes, NJ) < 30 min after collection and frozen at −80 °C until shipped overnight on dry ice for metabolomic analysis. The remaining semen was transferred to 50 mL polystyrene tubes and maintained at room temperature throughout semen quality analyses. Semen volume and pH were measured using a graduated cylinder and pH indicator strip (EM Science, Gibbstown, NJ), respectively. A freezing point depression osmometer (Advanced Instruments Inc., Norwood, MA) measured osmolality. Sperm concentration was calculated using cell counts from a hemacytometer (Hausser Scientific, Horsham, PA) after dilution with 6% saline solution (Montano Pedroso, 2010). Concentrations of samples collected in December 2024 were calculated using hemacytometer cell counts taken in June 2025 after freeze-thawing and dilution with 10% neutral-buffered formalin solution, due to availability of materials. Plasma membrane integrity was assessed with an eosin-nigrosin stain (Robeck & O’Brien, 2004). A minimum of 100 sperm were visually inspected under bright-field microscopy at 1000x magnification (Olympus BX51 microscope, Olympus Corporation, Hachioji, Tokyo, Japan). Sperm were classified as dead or live based on presence or absence of eosin stain uptake, respectively.

Semen was diluted to a concentration of 15–25 × 10^6^ sperm/mL using Androhep Enduraguard™ (Minitube of America, Verona, WI) for motility and kinematic analyses. An aliquot of 9 µL semen was pipetted onto a glass slide pre-warmed to 35 °C and covered with a 22 × 22 mm coverslip. Following a 2–3 min equilibration period, sperm were assessed using computer-aided semen analysis (CASA; HTM-IVOS Version 12.2; Hamilton Thorne, Inc., Beverly, MA). Sperm motility and kinematics were analyzed in phase contrast at 100x total magnification and 60 fps. Motility parameters assessed included total motility (%; average pathway velocity > 20 μm) and progressive motility (%; average pathway velocity > 50 μm/sec and straightness of movement > 80%). Kinematic parameters assessed included curvilinear velocity (VCL, µm/s), straight-line velocity (VSL, µm/s), average pathway velocity (VAP, µm/s), linearity index (LIN, %), straightness of movement (STR, %), amount of wobble (WOB, %), amplitude of lateral head displacement (ALH, µm), and beat cross frequency (BCF, Hz). A minimum of 200 sperm were analyzed for each semen sample across a minimum of five randomly selected microscopic fields.

### Semen thawing and subsampling

Semen samples were maintained in −80 °C storage for 130–170 days. Samples were removed from − 80 °C storage, held at −20 °C for ~ 24 h, then held at 4 °C for ~ 24 h to gradually temperature-adjust semen prior to processing. After thawing, all tubes were gently inverted three times and three 1 mL semen subsamples were collected. All tubes were then vortexed for 10 s to resuspend sperm and a second set of three 1 mL semen subsamples were collected. The first set of 1 mL subsamples were centrifuged at 4 °C for 10 min at 500 *g* to separate sperm from seminal plasma, yielding sperm-free seminal plasma for analyte analysis. Seminal plasma was visually inspected to verify the absence of sperm. The second set of 1 mL subsamples were not centrifuged and were used for whole semen analysis. Semen samples were processed in a randomized order.

### Analyte extraction

Metabolites were extracted from frozen-thawed semen according to modified procedures from Guinn et al. (2024). Aliquots of 400 µL pre-chilled 70:30 (v/v) acetonitrile:water (LC-MS Optima grade, Thermo Fisher Scientific, Waltham, MA) extraction solvent were combined with either 200 µL semen in lysing matrix tubes (1.4 mm Zirconium Beads, OPS Diagnostics, Lebanon, NJ) for semen analyte extraction or 200 µL sperm-free seminal plasma in microcentrifuge tubes for seminal plasma analyte extraction. Aliquots of 600 µL of extraction solvent were added to three lysing matrix tubes and three microcentrifuge tubes, which served as blank controls for each treatment. All tubes were vortexed at 3,200 *g* (Fixed Speed Vortex Mixer, VWR^®^, Radnor, PA) for 10 s. Sperm in lysing matrix tubes were homogenized in a bead beater (Precellys^®^ Evolution, Bertin Technologies, Montigny-le-Bretonneux, France) for 60 s, with 30 s intervals and a 30 s rest between cycles, at a speed of 5,000 *g*. After homogenization, lysing matrix tubes were vortexed for 10 s and all tubes were sonicated for 20 min at 30 °C (Branson M2800, Branson Ultrasonics™, Brookfield, CT). All tubes were centrifuged (Centrifuge 5424 R, Eppendorf, Hamburg, Germany) at 4 °C at 12,000 *g* for 10 min, and 350 µL supernatant was transferred to pre-chilled high-performance liquid chromatography (HPLC) vials. Fresh extraction solvent was added to the original lysing matrix and microcentrifuge tubes, and the homogenization, vortexing, sonicating, and centrifuging steps described above were repeated. An additional 350 µL aliquot of supernatant was transferred to the HPLC vials to give a final volume of 700 µL extracted solvent. Semen samples were diluted with chilled extraction solvent to a final concentration of 96 × 10^6^ sperm/mL. Analytes were extracted and analyzed in triplicate for each semen sample. Aliquots of 20 µL of each sample were combined to create a pooled quality control. Blank controls were created for semen and seminal plasma using the same respective extraction procedures without the samples. All extracts were stored at −20 °C until instrumental analysis.

### Instrumental analysis

Instrumental analysis followed modified procedures from Guinn et al. (2024). Semen and seminal plasma analytes were assessed with an ultra-high performance liquid chromatography (UHPLC) system (Vanquish, Thermo Fisher Scientific, Waltham, MA) coupled with an Orbitrap Fusion Tribrid mass spectrometer (Thermo Fisher Scientific, Waltham, MA) using an ACQUITY UPLC BEH C18 reversed-phase column (130 Å, 1.7 μm, 2.1 × 150 mm; Waters™ Corporation, Milford, MA). The mobile phases of the analysis employed UHPLC-grade Milli-Q water with 0.1% formic acid (eluent A) and UHPLC-grade acetonitrile with 0.1% formic acid (eluent B). Analyte separation occurred at a flow rate of 0.3 mL/min, with a 7 min re-equilibration period between samples. The mobile phase gradient used 5% eluent A and 95% eluent B from 0 to 2 min, ramped to 65% B for 18 min, ramped to 100% B for 1 min, and held at 100% B for 3 min. The UHPLC used an injection volume of 5 µL, and the Orbitrap full scan specified a mass range of 85–700 *m/z*. MS^2^ fragmentation scans were collected using the Quadrupole (0.7 *m/z* isolation window) with a stepped-energy collision-induced dissociation (CID) method (Ye et al., 2016). All samples and blank controls were run in positive and negative modes. All negative-mode analyses used a locking solution (85% CH_3_CN, 15% H_2_O, 0.08% NH_4_OH, 0.001% negative-locking solution) controlled by an external pump (Dionex AXP-MS, Thermo Fisher Scientific, Waltham, MA) at a flow rate of 0.1 mL/min. The pooled quality control was run every ten samples in both modes to quantify mass spectrometer performance over time.

### Untargeted analysis

Untargeted metabolite identification occurred using Compound Discoverer (v3.3) software (Thermo Fisher Scientific). Putative molecular compositions for each metabolite were generated using retention time, accurate mass measurements (mass error < 2 ppm), peak signal intensity (> 5,000), and MS^2^ fragmentation spectra. Detected features were matched against in-house mass databases generated in mzVault and external databases, including mzCloud (Thermo Fisher Scientific), Human Metabolome Database (HMDB; Wishart et al., 2007), Metabolika (Thermo Fisher Scientific), and ChemSpider (Pence & Williams, 2010). Each metabolite annotation was manually reviewed. Positive identifications were confirmed only when at least three MS^2^ fragment ions matched the fragmentation pattern of a corresponding pure analytical standard. Metabolite classes were assigned for compounds with significant differential expression between matrices and individuals using HMDB (Wishart et al., 2007), PubChem (Kim et al., 2025), LIPID MAPS (Liebisch et al., 2020), ClassyFire (Feunang et al., 2016), and ChEBI (Degtyarenko et al., 2008) databases. Any compounds not listed in the databases were labeled “Unknown.” Data normalization was conducted via the Systematic Error Removal using Random Forest (SERRF) algorithm to attenuate instrumental drift, inter-run discrepancies, and systematic technical biases. Intra- and inter-day precision were monitored through the injection of pooled quality control (QC) samples at intervals of 10 samples. SERRF employs a random forest architecture to capture and rectify complex, correlated systematic errors utilizing the variance derived from these QC injections. In alignment with our prior findings (Fan et al., 2019; Bergmann et al., 2024), this normalization effectively constrained technical variation, yielding an average relative standard deviation (RSD) of 5% and facilitating robust analytical reproducibility. Missing values were subsequently imputed using the post-alignment Fill Gaps function within Compound Discoverer software, applying a mass tolerance of 5 ppm and a signal-to-noise threshold of 1.5. Prior to further statistical evaluation, metabolites exhibiting missing values in 25% or more of the samples were discarded.

### Targeted analysis

A list of targeted hormone standards was selected based on the results of untargeted analysis and hormones identified in the semen/seminal plasma of terrestrial artiodactyls and humans in published literature (Supplemental Table 1). Hormone standards were purchased from Cayman Chemical (Ann Arbor, MI), Cerilliant (Round Rock, TX), Fisher Scientific (Hampton, NH), LGC Standards (Manchester, NH), and Sigma Aldrich (St. Louis, MO) and possessed ≥ 95% purity (Supplemental Tables 1 and 2). Non-labeled standards for the 27 selected hormones were dissolved in methanol and diluted to concentrations ranging from 0.0001 to 500 parts per billion (ppb) to generate calibration curves for quantification. Extraction performance was evaluated using three replicates of a single semen sample, a single seminal plasma sample, and three blanks which were spiked with known concentrations of isotopically labeled standards to achieve a final concentration of 20 ppb and processed according to the analyte extraction protocol (Sect. 2.4**)**. Recovery was calculated as the percentage of isotopically labeled hormone recovered following extraction. Precision was determined as the relative standard deviation of replicate recoveries. Data were processed using Skyline software (MacCoss Lab, University of Washington). Metabolite limits of detection (LOD) were determined based on reference standard retention times, peak signal intensities (> 1,000) with S/*N* > 3, and mass errors (< 3 ppm for positive mode without locking solution, < 2 ppm for negative mode with locking solution; Bergmann et al., 2024). Peaks with < 5 scans were designated as noise and excluded from subsequent analyses. Metabolite limits of quantification (LOQ) were determined based on LOD requirements and matching MS^2^ fragmentation to non-labeled analytical standards (Supplemental Table 1).

### Systems biology markup language model development

As explained in our recent study (Guinn et al., 2026), a genome-scale metabolic model for common bottlenose dolphins was constructed using an orthology-based reconstruction approach. The human-GEM served as the reference network. Protein sequences for common bottlenose dolphins were obtained in FASTA format from the NCBI RefSeq genome annotation (assembly mTurTru1) and processed through the CarveMe pipeline (Machado et al., 2018) to identify orthologs and transfer gene–protein–reaction associations from the human template to the dolphin genome, generating a species-specific SBML model. For metabolomic integration, experimental mass-to-charge (m/z) features were matched to the theoretical monoisotopic masses of metabolites contained within the dolphin SBML model. Matched metabolites were subsequently filtered using log₂ fold-change thresholds to identify significantly altered subsets. Pathway enrichment was evaluated using over-representation analysis with a hypergeometric test (Khatri & Drăghici, 2005; Marco-Ramell et al., 2018), with pathway definitions derived directly from the metabolic network structure. Statistical significance was adjusted using the Benjamini–Hochberg procedure to control the false discovery rate (FDR).

### Data analyses

Principal component analyses were generated in Compound Discoverer (v3.3) to compare metabolome profile variance between biological matrices (semen and seminal plasma), individuals, and collection dates. Permutational multivariate analysis of variance (PERMANOVA) were computed on PC scores in R statistical software (v4.5.2; R Core Team, 2023) using Euclidean distance and 999 permutations in the vegan package (Oksanen et al., 2026). Pairwise comparison *p*-values were adjusted using the Bonferroni correction. Permutations were constrained within individuals for the biological matrix analysis and multivariate dispersion homogeneity was verified using the betadisper function. Volcano plots were created in Compound Discoverer to compare metabolite enrichment variation across biological matrices, individuals, and collection dates. Male 3 was excluded from volcano plot comparisons due to lack of biological sample replicates. Metabolite enrichment differences between biological matrices and individuals were assessed using t-tests with Benjamini-Hochberg adjustment method to control for false discovery rate. Repeated measures correlations were computed in R between sperm quality parameters and the normalized peak areas of all metabolites detected in the untargeted analysis. The rmcorr package (Bakdash & Marusich, 2026) was used and *p*-values were adjusted using the Benjamin-Hochberg false discovery rate correction. Among the metabolites with significant correlations to semen quality parameters, identities were reported only for metabolites with MS^2^ fragmentation spectra with a mzVault/mzCloud match confidence or FiSH score of ≥ 50 for metabolites with a coeluting metabolite within 0.7 Da and ≥ 70 for metabolites without a coeluting metabolite within 0.7 Da. Metabolomic pathway analyses were conducted in Python software (v3.12; Python Software Foundation, 2026) using a dolphin metabolite SBML model developed for this study (Sect. 2.8). Data visualizations were generated in R statistical software.

## Results

### Untargeted analysis

**Fig. 2:**
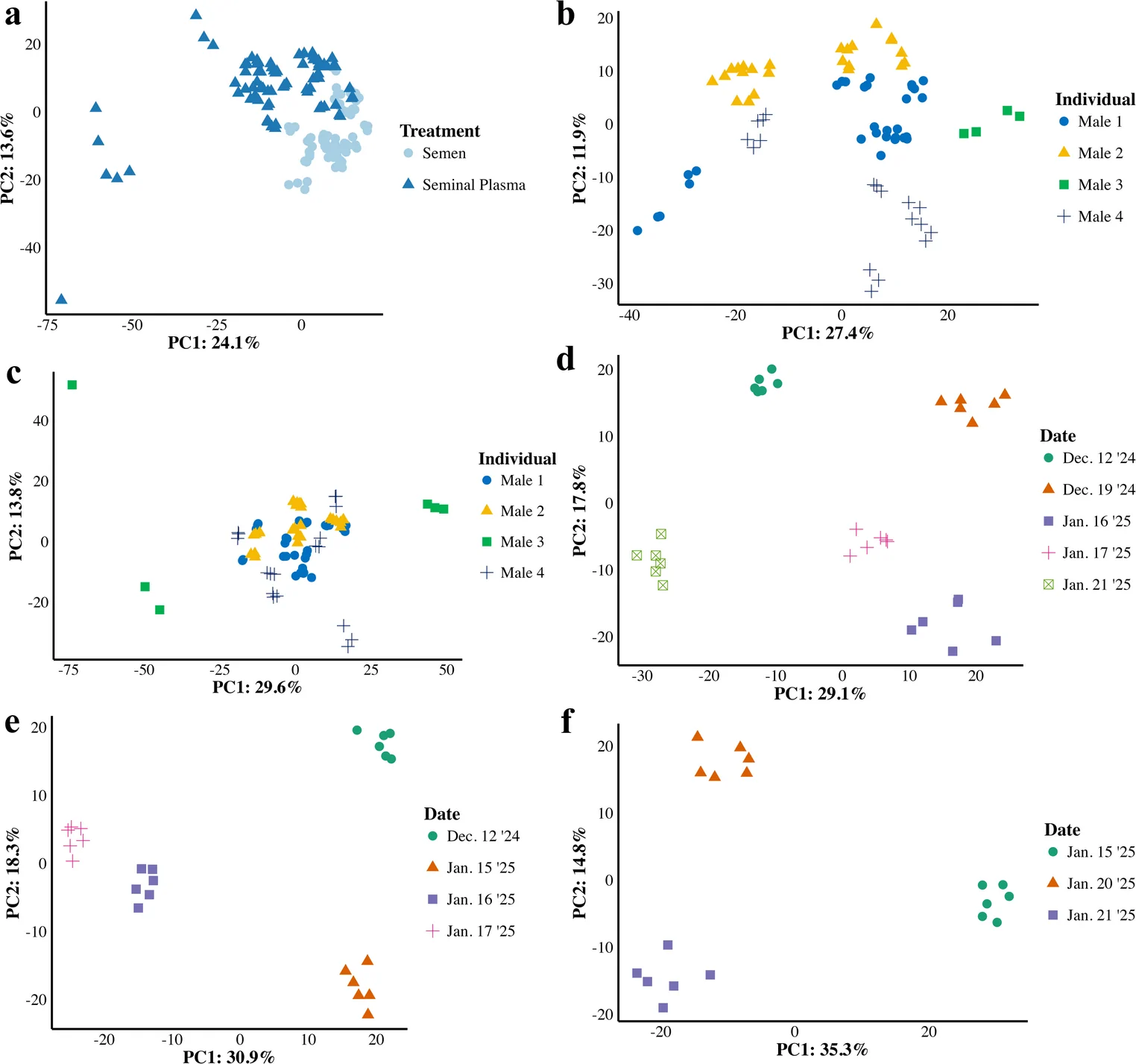
Principal component analyses of metabolite variability of four common bottlenose dolphins (*Tursiops truncatus*) attributable to: **a** biological matrix (semen and seminal plasma), **b** individual (semen only), **c** individual (seminal plasma only), **d** collection date for Male 1 (seminal plasma only), **e** collection date for Male 2 (seminal plasma only), and **f** collection date for Male 4 (seminal plasma only). *n* = 2,198 metabolites.

Untargeted analysis detected 2,198 metabolites across dolphin semen and seminal plasma samples. Principal component analyses revealed visual separation of metabolite profiles between semen and seminal plasma (Fig. 2a), individuals (Fig. 2b-c), and temporally within individuals (Fig. 2d-f and Supplemental Fig. 1a-c). PERMANOVA analyses indicated significant differences between semen and seminal plasma metabolite profiles (F = 36.9, R^2^ = 0.33, *p* = 0.001). Semen and seminal plasma were also significantly different in multivariate dispersion (F = 6.6, *p* = 0.007). Semen metabolite profiles significantly differed between individuals (F = 6.32, R^2^ = 0.36, *p* = 0.001), with significant separation between Males 2 and 4 (F = 15.11, R^2^ = 0.44, *p* = 0.01) and Males 2 and 3 (F = 13.83, R^2^ = 0.54, *p* = 0.04). Seminal plasma metabolite profiles also significantly differed between individuals (F = 15.32, R^2^ = 0.57, *p* = 0.001), with significant differences between Males 1 and 3 (F = 39.56, R^2^ = 0.71, *p* = 0.01), Males 2 and 3 (F = 37.37, R^2^ = 0.74, *p* = 0.01), and Males 4 and 2 (F = 11.83, R^2^ = 0.54, *p* = 0.02). Seminal plasma samples were significantly different in multivariate dispersion (F = 2.9, *p* = 0.048). While the collection of only one semen sample from Male 3 precluded temporal analyses, significant temporal differences in metabolite profiles were observed in the seminal plasma and semen of Male 1 (seminal plasma: F = 262.44, R^2^ = 0.99, *p* = 0.001; semen: F = 151.71, R^2^ = 0.98, *p* = 0.001), Male 2 (seminal plasma: F = 283.84, R^2^ = 0.99, *p* = 0.001; semen: F = 109.51, R^2^ = 0.98, *p* = 0.001), and Male 4 (seminal plasma: F = 293.49, R^2^ = 0.99, *p* = 0.004; semen: F = 757.65, R^2^ = 1, *p* = 0.004), indicating potential time-dependent variation in metabolite composition.

**Fig. 3:**
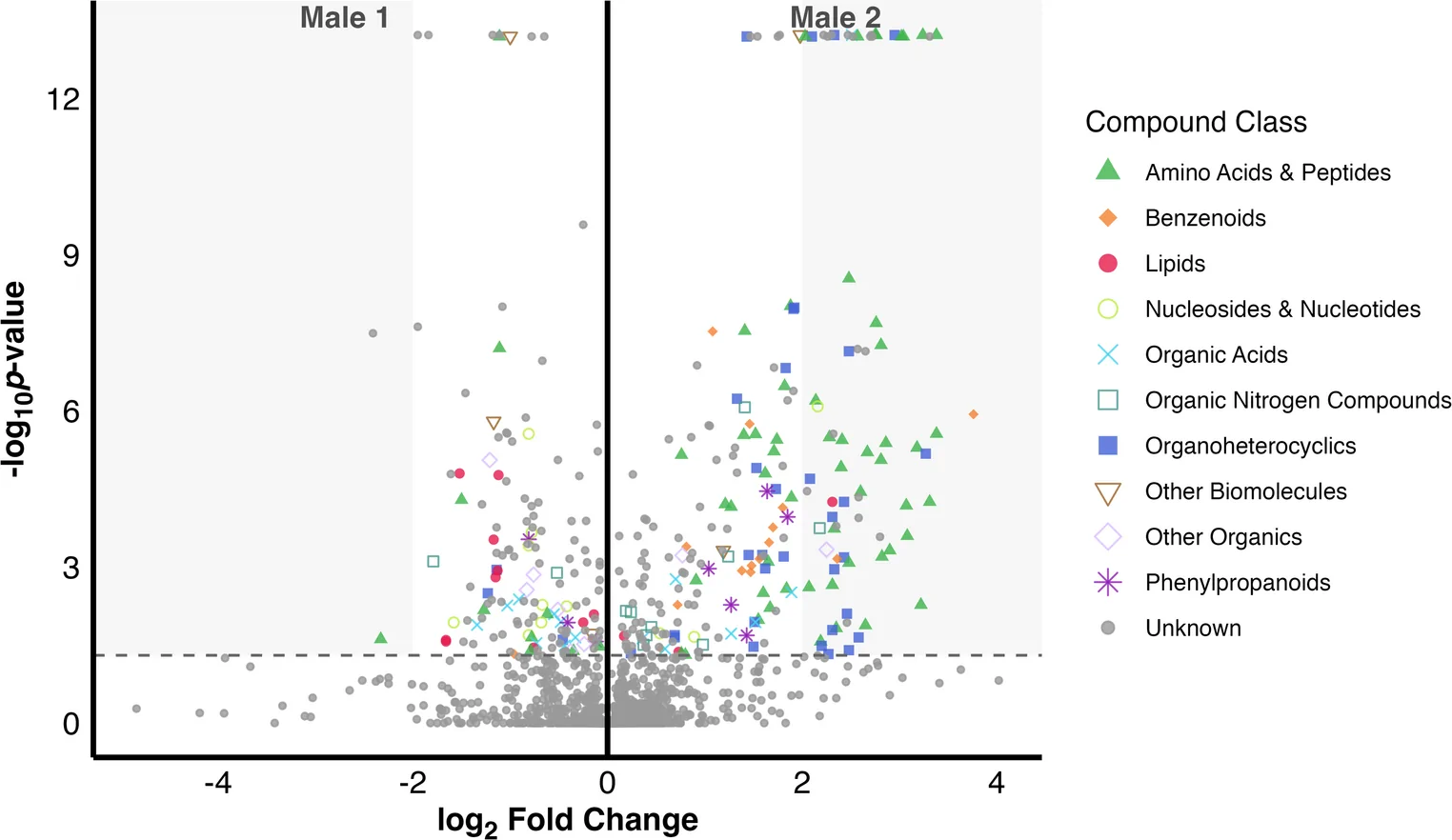
Volcano plot of differential semen metabolite abundance between common bottlenose dolphins (*Tursiops truncatus*) Male 1 and 2. The x-axis represents the log_2_ fold change in metabolite abundance and the y-axis represents the -log_10_ of adjusted *p*-values. All points on or above the dashed line have a *p*-value of ≤ 0.05 and all points within the light grey shaded region have a *p*-value of ≤ 0.05 and log_2_ fold change ≥ 2. Metabolite classes are indicated by point shape and color. *n* = 2,198 metabolites.

The relative abundances of 353 metabolites differed significantly between semen and seminal plasma (*p* < 0.05), with 101 and 252 metabolites significantly enriched in semen and seminal plasma, respectively (*p* < 0.05; log_2_ fold ≥ 1). Both semen and seminal plasma were significantly enriched in lipids and organoheterocyclic compounds, while semen had higher enrichment of benzenoids and seminal plasma had higher enrichment of amino acids and peptides. Although seminal plasma contained a higher total number of significantly enriched metabolites, semen contained a higher number of metabolites with a log_2_ fold change ≥ 2, representing a stronger degree of metabolite enrichment (Supplemental Fig. 2). Across all semen samples, amino acids, peptides, and organoheterocyclics were the most frequently enriched metabolite classes (Fig. 3 & Supplemental Figs. 3–4). The extent of metabolite enrichment in semen varied between individuals; Male 3 exhibited the highest number of upregulated semen metabolites and Male 4 exhibited the fewest (Supplemental Table 3). The semen of Males 1 and 4 were enriched with phenylpropanoids (Fig. 3; Supplemental Figs. 3–4) while the semen of Male 2 was enriched with benzenoids (Fig. 3; Supplemental Fig. 4).

### Targeted analysis

**Fig. 4:**
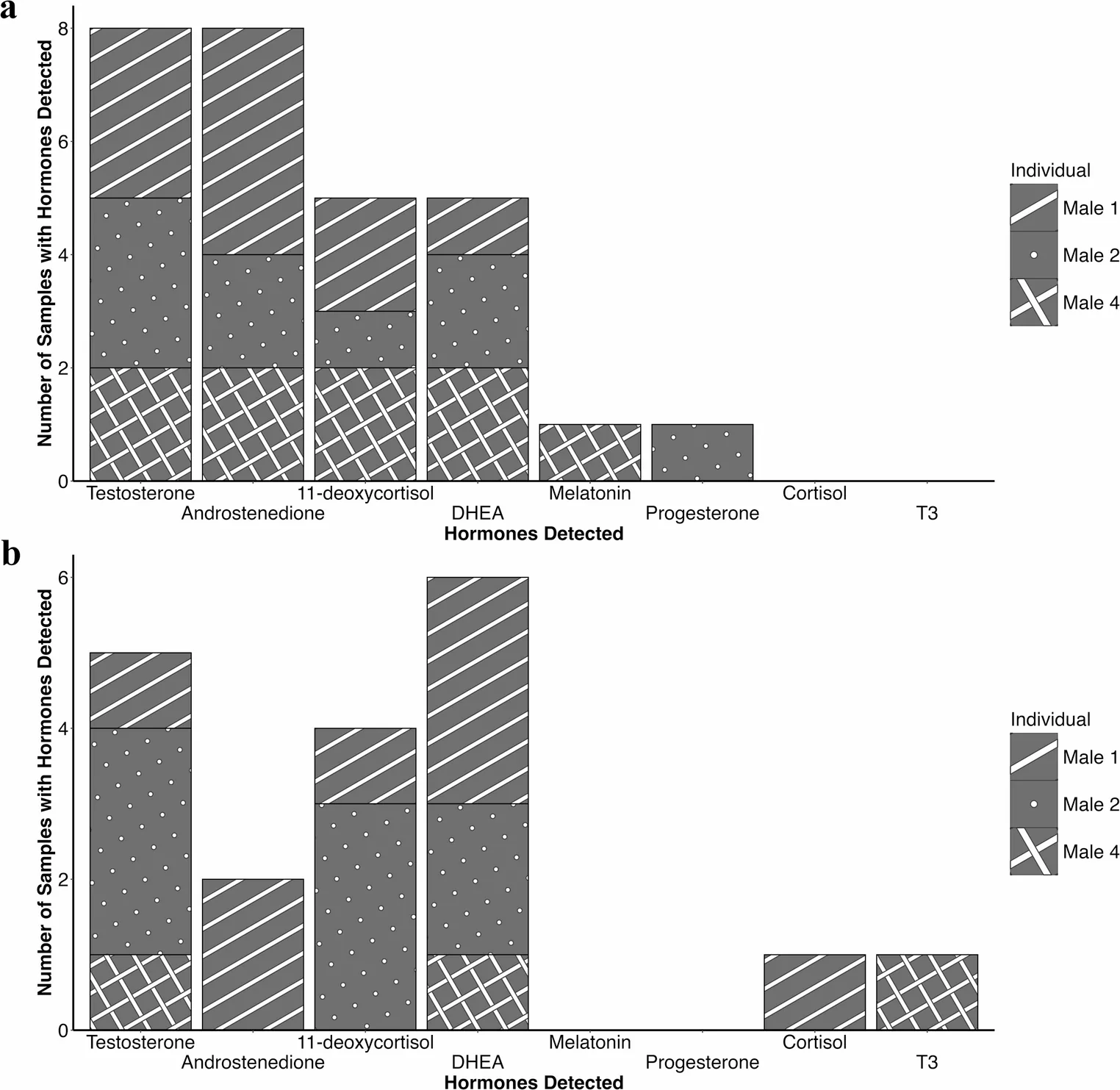
Hormone detections in common bottlenose dolphin (*Tursiops truncatus*): **a** semen and **b** seminal plasma. *n* = 12 ejaculates. Bar patterns denote detections within individuals.

Targeted analysis confirmed detection of eight hormones across dolphin semen (Fig. 4a) and seminal plasma (Fig. 4b). One non-reproductive and five reproductive hormones were identified in semen, while six reproductive hormones were identified in seminal plasma (Fig. 4). No hormones were detected in Male 3. All detected hormones were above the limit of detection but below the limit of quantification, precluding calculation of hormone concentrations. Testosterone was detected in eight semen and five seminal plasma samples from Males 1, 2, and 4. Androstenedione was detected in eight semen samples from Males 1, 2, and 4 and in two seminal plasma samples from Male 1. Dehydroepiandrosterone (DHEA) was detected in five semen and six seminal plasma samples from Males 1, 2, and 4. 11-deoxycortisol was detected in five semen samples from Males 1, 2, and 4 and four seminal plasma samples from Males 1 and 2. Progesterone and melatonin were each detected in a single semen sample from Male 2 and Male 4, respectively. In seminal plasma, cortisol was detected in one sample from Male 1. 3,3,5-Triiodo-L-thyronine (T3) was detected in one sample from Male 4.

### Metabolomic pathway enrichment analysis

**Fig. 5:**
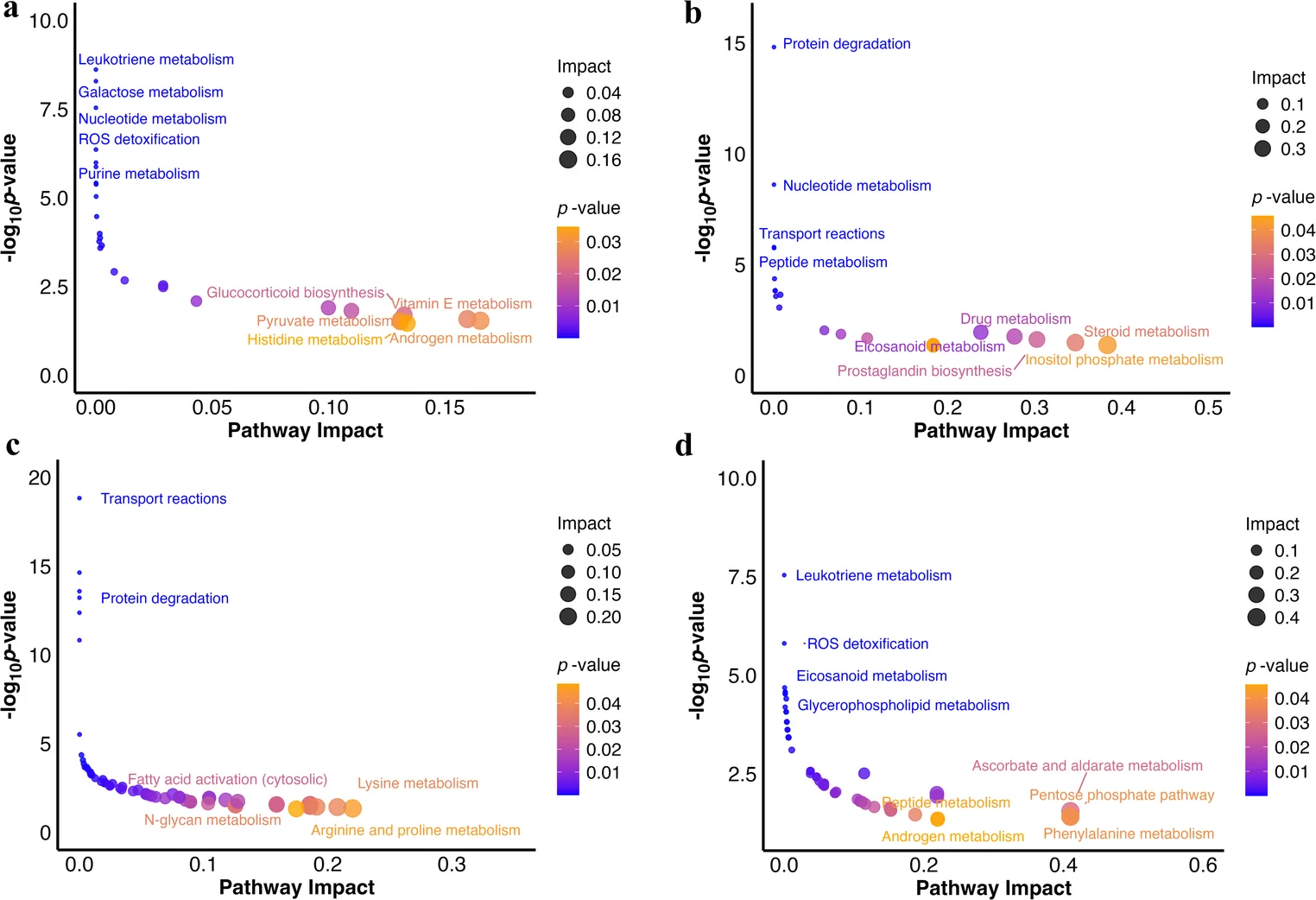
Pathway enrichment analysis of metabolite variability of common bottlenose dolphin (*Tursiops truncatus*) semen among **a** Male 1, **b** Male 2, **c** Male 3, and **d** Male 4. Point size indicates the strength of the pathway impact and point color indicates the pathway enrichment significance. The five most significantly enriched and highest impact pathways are labeled for each individual. *n* = 2,198 metabolites.

Pathway analysis using the dolphin SBML metabolic model revealed distinct patterns of pathway enrichment across the four individuals (Fig. 5). Males 1 (*p* = 2.91 × 10^− 8^, pathway impact = 8.44 × 10^− 7^) and 2 (*p* = 2.49 × 10^− 9^, pathway impact = 6.97 × 10^− 8^) had significant enrichment of nucleotide metabolism pathways. Males 2 and 3 possessed significant enrichment of protein degradation (Male 2: *p* = 1.57 × 10^− 15^, pathway impact = 8.79 × 10^− 14^; Male 3: *p* = 6.11 × 10^− 14^, pathway impact = 2.97 × 10^− 12^) and transport reaction (Male 2: *p* = 1.65 × 10^− 6^, pathway impact = 3.07 × 10^− 5^; Male 3: *p* = 1.48 × 10^− 19^, pathway impact = 1.60 × 10^− 17^) pathways. Male 2 had significant enrichment of peptide metabolism pathway (*p* = 1.80 × 10^− 6^, pathway impact = 1.13 × 10^− 4^) and significant pathway impact of eicosanoid metabolism (*p* = 0.01, pathway impact = 0.24), while Male 4 had significant enrichment of eicosanoid metabolism pathway (*p* = 2.07 × 10^− 5^, pathway impact = 5.66 × 10^− 4^) and significant pathway impact of peptide metabolism (*p* = 0.04, pathway impact = 0.22). Males 1 and 4 exhibited significant enrichment of leukotriene metabolism (Male 1: *p* = 2.44 × 10^− 9^, pathway impact = 1.93 × 10^− 7^; Male 4: *p* = 2.89 × 10^− 8^, pathway impact = 2.37 × 10^− 6^) and reactive oxygen species (ROS) detoxification pathways (Male 1: *p* = 4.38 × 10^− 7^, pathway impact = 1.73 × 10^− 5^; Male 4: *p* = 1.56 × 10^− 6^, pathway impact = 6.39 × 10^− 5^) and had high pathway impacts from androgen metabolism pathways (Male 1: *p* = 0.03, pathway impact = 0.17; Male 4: *p* = 0.05, pathway impact = 0.22).

### Metabolite correlations with semen quality parameters

All semen samples exhibited quality parameters within the normal range for this species (Table 2; van der Horst et al., 2018). Among the 2,198 metabolites detected, 98 exhibited significant correlations with semen quality parameters (*p* < 0.0005). Sperm concentration was the parameter most frequently correlated with metabolite abundance (82 metabolites), followed by osmolality (12 metabolites). Seven of the 98 correlating metabolites were tentatively identified by matching mzCloud MS/MS database or *in silico* fragmentation (Table 3). Among these identified metabolites, six were positively correlated with semen quality metrics, whereas trimethylamine N-oxide positively correlated with osmolality but negatively correlated with straight-line velocity and average pathway velocity (Table 3). Organic acids represented the metabolite superclass most frequently correlated with semen quality parameters.

**Table 2:** Semen quality parameters of four common bottlenose dolphins (*Tursiops truncatus*) across collection dates.

Collection date	Male	Vol	pH	Osmo	Conc	Viab	TM/PM	VAP	VSL/VCL	STR/LIN	ALH	BCF	WOB
12/12/24	Male 1 Male 2 Male 3	> 100 10 10	7.1 7.4 7.4	334 334 348	261 388 1710	- - -	88/85 97/92 89/81	181 156 167	181/238 156/264 168/256	95/78 93/61 90/68	5.4 9.0 7.1	42 34 40	81 64 73
12/19/24	Male 1	> 100	7.1	335	476	-	93/92	177	169/221	92/74	4.9	40	77
01/15/25	Male 2 Male 4	30 107	6.8 7.1	336 338	269 99	- -	97/97 86/81	165 152	157/223 145/210	93/71 85/63	6.4 5.6	38 39	74 67
01/16/25	Male 1 Male 2	51 18	6.8 7.1	349 336	472 148	93 100	87/85 93/92	148 160	142/187 150/224	85/68 89/65	4.7 6.5	36 36	71 70
01/17/25	Male 1 Male 2	21 18	7.1 7.1	334 339	368 156	95 100	95/94 96/94	185 150	179/230 138/221	92/75 90/62	5.1 7.6	41 33	77 68
01/20/25	Male 4	75	7.4	337	297	98	77/77	138	128/195	75/55	5.5	31	59
01/21/25	Male 1	100	7.4	339	96	92	88/82	170	157/228	85/66	5.9	38	71

Dashed lines indicate quality metrics were not collected for the individual on the specified date.

Quality parameter abbreviations and units: Vol = volume (mL), Osmo = osmolality (mOsm), Conc = sperm concentration (x10^6^/mL), Viab = sperm viability (%), TM = total motility (%), PM = progressive motility (%), VAP = average pathway velocity (µm/s), VSL = straight-line velocity (µm/s), VCL = curvilinear velocity (µm/s), STR = straightness of movement (%), LIN = linearity index (%), ALH = amplitude of lateral head displacement (µm), BCF = beat cross frequency (Hz), WOB = amount of wobble (%).

**Table 3:** Common bottlenose dolphin (*Tursiops truncatus*) semen and seminal plasma metabolites correlated with semen quality parameters.

Metabolite	Correlated semen quality parameter	Positive (+)/negative (-) correlation	*R* ^2^	Degrees of freedom	*p*-value
1-Methylhistidine	Concentration	+	0.75	19	9.68 × 10^− 5^
Galactonic acid	Concentration	+	0.74	19	1.19 × 10^− 4^
Glycerophosphoinositol	Concentration	+	0.79	19	1.72 × 10^− 5^
Malic acid	Viability	+	0.93	8	7.46 × 10^− 5^
Trimethylamine N-oxide	Osmolality VSL VAP	+ - -	0.82 0.78 0.74	19 19 19	6.44 × 10^− 6^ 3.75 × 10^− 5^ 1.43 × 10^− 4^
Uric acid	Osmolality	+	0.85	19	9.51 × 10^− 7^
Xanthine	Concentration	+	0.74	19	1.22 × 10^− 4^

VSL = straight-line velocity (µm/s), VAP = average pathway velocity (µm/s).

## Discussion

Untargeted metabolomic analysis identified 2,198 distinct metabolites in the semen and seminal plasma of common bottlenose dolphins, revealing substantial biochemical complexity within these biological matrices. Metabolome profiles varied across sample type, individuals, and collection dates, while pathway enrichment analyses highlighted distinct individual metabolic patterns. Significant associations between several metabolites and semen quality parameters suggest that underlying metabolic processes may contribute to variability in reproductive health and may serve as potential indicators of semen quality in common bottlenose dolphins.

### Untargeted analyses of metabolome profiles

Metabolome profiles of both biological matrices were significantly enriched in lipids and organoheterocyclic compounds. Seminal plasma was significantly enriched in amino acids and peptides while semen was significantly enriched in benzenoids. As in humans, variability in biological matrix metabolites is likely due to differences in metabolite concentrations that modulate sperm behavior and protect sperm in semen and seminal plasma (Engel et al., 2019). Metabolome profiles varied significantly between individual dolphins, as observed in swine (Mateo-Otero et al., 2020; Dlamini et al., 2025), with greater metabolite variability at the individual level in semen than in seminal plasma. Individual identity may have affected semen metabolite variability to a greater extent due to genetic effects on individual sperm metabolomes (Engel et al., 2019), variable dilution factors (Engel et al., 2019), freeze-thawing effects on semen metabolome (Longobardi et al., 2020), age (Beg et al., 2026), or fertility status of individuals (Peluso et al., 2025). Analysis of additional semen samples and genetic analyses may clarify the genotypic and phenotypic mechanisms controlling individual semen metabolite regulation. Semen and seminal plasma metabolites showed significant temporal variability within individuals. As bottlenose dolphins experience seasonal peaks in sperm production during summer and fall (Schroeder & Keller, 1989; O’Brien & Robeck, 2010) and semen was collected over six weeks in winter, strong temporal variability in metabolites was not expected. Temporal clustering may be driven by semen collection frequency as increased frequencies altered metabolomic profiles of human semen (Alipour et al., 2021). Collection of samples over longer time periods may elucidate the role of specific metabolites in seasonal spermatogenesis patterns of bottlenose dolphins.

### Targeted hormone analyses

The detection of the reproductive hormone testosterone and its precursors, androstenedione and dehydroepiandrosterone, was expected as they control spermatogenesis across species (Vitku et al., 2017; Guvvala et al., 2020). As serum testosterone concentrations peak in spring and summer in bottlenose dolphins (Schroeder & Keller, 1989), pronounced variability in testosterone detection and testosterone-mediated semen quality parameters were not expected in semen samples collected in winter. Cortisol, a steroid hormone that regulates androgen biosynthesis in human and domestic ruminant seminal plasma (Hampl et al., 2013; Guvvala et al., 2020), was detected less frequently than its precursor hormone, 11-deoxycortisol (Slominski et al., 2020). 3,3,5-Triiodo-L-thyronine (T3) modulates sperm concentrations in mammals (Eğritağ et al., 2022) and was detected in seminal plasma at low frequencies in dolphins. Low cortisol and T3 frequencies may promote increased androgen biosynthesis and spermatogenesis in common bottlenose dolphins that have weak reproductive seasonality (O’Brien & Robeck, 2010). Progesterone is found in seminal plasma in concentrations similar to serum and modulates motility and the acrosome reaction in human sperm (Modi et al., 2007; Vitku et al., 2017). Progesterone was detected with low frequencies, possibly suggesting reduced functionality in bottlenose dolphin semen. Specific concentrations of progesterone in dolphin seminal plasma and reported effects have not been previously published. Although an analytical standard for estradiol was undetectable, the presence of estradiol in dolphin semen is expected as it is present in the semen of closely related artiodactyl species (Guvvala et al., 2020).

Melatonin, the only non-reproductive hormone detected in a single semen sample from Male 4, acts as an antioxidant in mammalian semen and improves semen quality (Vitku et al., 2017). Male 4 possessed significantly enriched pathways for ROS detoxification, suggesting an active antioxidant role. Melatonin detection in bottlenose dolphin semen was unexpected as cetaceans do not possess functional genes for melatonin synthesis to facilitate unihemispheric sleep (Huelsmann et al., 2019), suggesting dietary, potential extrapineal production, or alternative origin. Analyses of additional semen samples and prey items may elucidate the origin, half-life, and function of melatonin in cetacean semen. The absence of detected hormones and the lack of significant enrichment in androgen and steroid metabolism pathways in Male 3 may indicate interruption of steroid synthesis pathways, which could alter reproductive function. Variable hormone detection rates between semen and seminal plasma, and across individuals, may be attributable to differences in metabolomic pathway enrichment as observed in humans (Lu et al., 2026).

### Metabolomic pathway analyses

Pathway analysis using an SBML model based on common bottlenose dolphin genetics identified differential enrichment of metabolic pathways across individuals. High impact of androgen metabolic pathways in Males 1 and 4 supports the high frequencies of androgen detection. Significant enrichment of leukotriene (inflammatory mediary metabolites) metabolism and ROS detoxification pathways in Males 1 and 4 suggests oxidative stress occurred in their semen (Dias et al., 2019). High pathway impact of metabolism of additional metabolites that function as antioxidants in semen was noted in Males 1 and 4, such as vitamin E, eicosanoid, and phenylalanine (Bansal & Bilaspuri, 2010). The enrichment of phenylpropanoids in the semen of Males 1 and 4 may also relate to the upregulation of ROS detoxification as phenylpropanoids can function as antioxidants (Kostyunina et al., 2025). PCA clustering of metabolites from Male 1 and 4 may relate to similar pathway enrichment in antioxidant metabolism and ROS detoxification. High pathway impact of glucocorticoid biosynthesis and detection of cortisol in the seminal plasma of Male 1 may also be related to oxidative stress (Darbandi et al., 2018). The causal agent for potential oxidative stress in the semen of Males 1 and 4 remains unclear as all dolphins were considered clinically healthy and were not being treated for any injuries or infections during the semen collection period.

Despite possible oxidative stress in some individuals, semen samples collected from all four individuals possessed semen quality parameters within normal species ranges for high-quality semen (Yuen et al., 2009; van der Horst et al., 2018). Pathways associated with amino acid metabolism and amino acid abundances were enriched across individuals, with variation between individuals in the specific amino acids enriched. High pathway impact of amino acid (lysine, arginine, and proline) and n-glycan metabolism in Male 3 may indicate metabolic investment in sperm motility (Engel et al., 2019). Enrichment of galactose and high impact of pyruvate pathways in Male 1 may also be associated with maintaining sperm motility, as found in bulls (Menezes et al., 2019). Prostaglandin biosynthesis and fatty acid activation, which support sperm transport and fertilization in humans, had significant pathway impacts in Males 2 and 3, respectively (Gottlieb et al., 1988; Collodel et al., 2020). Significant enrichment of nucleotide metabolism pathways in Males 1 and 2 and peptide metabolism pathways in Male 2 may relate to semen quality parameters, as has been identified in swine (Zhang et al., 2024); however, proteomic analyses would be necessary to inform species-specific relationships. Although all dolphins received dietary supplements appropriate to their body weight, provisioned vitamins may have affected metabolomic pathway enrichment. Metabolomic assessments of individuals with low-quality semen parameters could further elucidate the species-specific metabolomic pathways involved in maintaining high semen quality in dolphins and provide deeper analytical tools for assessing semen quality than current quality parameters (Castiglione Morelli et al., 2021).

### Metabolite correlations with semen quality

Although all detected hormones were below the limit of quantification and could not be correlated with semen quality, non-hormone metabolite correlations with quality parameters identified potential novel biomarkers of dolphin semen quality. Although a correlation between 1-Methylhistidine and sperm concentration has not been documented in other species, 1-Methylhistidine has been identified in the seminal plasma of Japanese quail (*Coturnix japonica*) and has been shown to function alongside other amino acids as an antioxidant in semen (Sarkar et al., 2021; Wu et al., 2003). Xanthine has been identified as a biomarker of altered fertility in humans (Qiao et al., 2017); however, the mechanistic relationship between xanthine and semen quality parameters is unclear. As increased oxidation of xanthine by xanthine oxidase reduces spermatogenesis in humans (Medubi et al., 2025), the positive correlation between xanthine and sperm concentration in dolphins may indicate reduced levels of xanthine oxidation and ROS. While no studies have directly reported correlations between glycerophosphoinositol and sperm concentration, other glycerophospholipids, such as glycerophosphocoline, positively correlate with sperm concentration in humans (Mieusset et al., 1988). Based on the high levels of glycerophospholipids in dolphin semen and the function of glycerophospholipids in maintaining sperm membrane integrity in other mammals, it is hypothesized that increased glycerophosphoinositol in dolphin semen may increase sperm concentration by supporting spermatogenesis though cell membrane development (Alves et al., 2021; Shan et al., 2021). A relationship between galactonic acid and sperm concentration has not been identified in other species. Additionally, relationships between trimethylamine N-oxide nor uric acid and semen osmolality have not been established in any other species. Endogenous malic acid has not been directly correlated with sperm viability in any species. Malic acid concentrations were reduced in the semen of humans with oligospermia and azoospermia (Saraf et al., 2020; Arslan et al., 2023), indirectly associating malic acid with sperm viability through concentration metrics. Negative correlations between trimethylamine N-oxide and sperm motility in dolphins match patterns of reduced motility with increased blood serum levels of trimethylamine N-oxide in humans (Wang et al., 2026). Increased oxidative stress is hypothesized to result from increased trimethylamine N-oxide levels, thereby reducing semen quality (Wang et al., 2026).

### Limitations

Future analyses may address study limitations, including absence of validation cohort, small sample size, reliance on frozen-thawed semen, and inability to relate metabolome variability to realized fertility. The absence of a validation cohort and limited sample size were a result of the limited number of dolphins trained for voluntary semen collection and inconsistent collection success among trained individuals. The absence of biological replicates and high dilution factor of Male 3’s semen may have affected hormone detection rates for this individual and skewed metabolome profile comparisons with other males. Reliance on frozen-thawed semen may have affected biological matrix metabolome profiles as sperm-specific metabolites may be introduced into the sperm-free seminal plasma during freeze-thawing (Hammadeh et al., 1999). Limited sample size and parentage history of the males included in the study prohibited analysis of metabolome profiles and fertility. Further research exploration of the proposed semen quality biomarkers incorporating fertilization success rates is warranted.

## Conclusions

This study provides the first metabolome profile of semen and seminal plasma of any marine mammal species. The study findings provide critical baseline data on the drivers of variability in the metabolome profiles of semen and seminal plasma of common bottlenose dolphins and present potential novel metabolomic markers of semen quality. The SBML model used in this study has been made openly available and has potential applications in metabolomic analyses across tissue matrices, sexes, age classes, and populations. Metabolomic comparisons between fresh and frozen-thawed dolphin semen may validate results and identify species-specific markers of freezability for cryopreservation as accomplished for multiple livestock species (Zhang et al., 2021; Sui et al., 2023; Xu et al., 2023). Future analyses of semen samples from several artiodactyl species could elucidate conserved metabolites associated with semen quality.

## Supplementary Information

Below is the link to the electronic supplementary material.

Supplementary Material 1

## Data Availability

The metabolomics and metadata reported in this paper are available via https://www.ebi.ac.uk/metabolights/MTBLS15902 study identifier MTBLS15902.

## Abbreviations

ART: Assisted reproductive technologies
CASA: Computer-aided semen analysis
Da: dalton
HPLC: High-performance liquid chromatography
QC: Quality control
ROS: Reactive oxygen species
SERF: Systematic Error Removal using Random Forest
SBML: Systems biology markup language
UHPLC: Ultra-high performance liquid chromatography

## Code availability

The code for the SBML model of dolphin metabolites used in this study is available via https://doi.org/10.5281/zenodo.20947629.
