# Supplementary material for: Dolphin semen metabolome characterization and relationships with sperm quality
Source: Metabolomics. 2026 Nov 13;22(6):177. doi: 10.1007/s11306-026-02552-z (PMC13768358; doi:10.1007/s11306-026-02552-z)
Supplement: Supplementary file 1 [file 11306_2026_2552_MOESM1_ESM.docx]

Supplemental Table 1. Non-labeled hormone standards and manufacturers

| **Non-labeled standard** | **Manufacturer** |
| --- | --- |
| 11-Deoxycorticosterone | Fisher Scientific |
| 11-Deoxycortisol | Fisher Scientific |
| 17alpha hydroxyprogesterone | Fisher Scientific |
| 3,3’,5-Triiodo-L-thyronine | Cayman Chemical |
| (5a)-androst-16-en-3-one | Cayman Chemical |
| 5alpha-Dihydrotestosterone | Cerilliant |
| Aldosterone | Fisher Scientific |
| Androstenedione | Fisher Scientific |
| Corticosterone | Fisher Scientific |
| Cortisol | Fisher Scientific |
| Cortisone | Fisher Scientific |
| Dehydroepiandrosterone | Cayman Chemical |
| Dehydroepiandrosterone-Sulfate | Cayman Chemical |
| Epinephrine | Sigma Aldrich |
| Estradiol | Fisher Scientific |
| Estriol | Cayman Chemical |
| Estrone | Cayman Chemical |
| L-Thyroxine | Cayman Chemical |
| Lysipressin | Cayman Chemical |
| Melatonin | Cayman Chemical |
| Norepinephrine | Cayman Chemical |
| Oxytocin | Cayman Chemical |
| Pregnenolone | Cayman Chemical |
| Progesterone | Fisher Scientific |
| Testosterone | Fisher Scientific |

Supplemental Table 2. Isotopically labeled hormone standards and manufacturers

| **Isotopically labeled standard** | **Manufacturer** |
| --- | --- |
| 17alpha hydroxyprogesterone-^13^C_3_ | Fisher Scientific |
| 16-(5a)-Androsten-3-one-2,2,4,4-D_4_ | LGC Standards |
| 5alpha-Dihydrotestosterone-16,16,17-D_3_ | Cerilliant |
| Aldosterone-^13^C_3_ | Fisher Scientific |
| Androstenedione-^13^C_3_ | Fisher Scientific |
| Corticosterone-^13^C_3_ | Fisher Scientific |
| Cortisol-^13^C_3_ | Fisher Scientific |
| Cortisone-^13^C_3_ | Fisher Scientific |
| Epinephrine-D_3_ | Cayman Chemical |
| Estradiol-^13^C_3_ | Fisher Scientific |
| Estriol-D_2_ | Cayman Chemical |
| Estrone-D_2_ | Cayman Chemical |
| Melatonin-D_4_ | Cayman Chemical |
| Pregnenolone-20,21-^13^C_2_-16,16-D_2_ | Sigma Aldrich |
| Progesterone-^13^C_3_ | Fisher Scientific |
| Testosterone-^13^C_3_ | Fisher Scientific |

Supplemental Table 3. Number of metabolites significantly upregulated in individual common bottlenose dolphins’ (*Tursiops truncatus*) semen across rows compared to individuals down columns. n = 2,198 metabolites. *p* < 0.05 for all metabolites.

|  | **Male 1** | **Male 2** | **Male 3** | **Male 4** |
| --- | --- | --- | --- | --- |
| **Male 1** |  | 27 upregulated | 85 upregulated | 8 upregulated |
| **Male 2** | 92 upregulated |  | 68 upregulated | 93 upregulated |
| **Male 3** | 217 upregulated | 43 upregulated |  | 194 upregulated |
| **Male 4** | 12 upregulated | 52 upregulated | 53 upregulated |  |

Supplemental Figure 1. Principle component analysis of temporal variability in semen metabolites in a) Male 1 b) Male 2, and c) Male 4 common bottlenose dolphins (*Tursiops truncatus*). n = 2,198 metabolites.

Supplemental Figure 2. Volcano plot of differential semen and seminal plasma metabolite abundance of four common bottlenose dolphins (*Tursiops truncatus*). The x-axis represents the log_2_ fold change in metabolite abundance and the y-axis represents the -log_10_ of adjusted *p*-values. All points on or above the dashed line have a *p*-value of ≤ 0.05 and all points within the light grey shaded region have a *p*-value of ≤ 0.05 and log_2_ fold change ≥ 2. Metabolite classes are indicated by point shape and color. n = 2,198 metabolites.

Supplemental Figure 3. Volcano plot of differential semen metabolite abundance between common bottlenose dolphins (*Tursiops truncatus*) Male 1 and 4. The x-axis represents the log_2_ fold change in metabolite abundance and the y-axis represents the -log_10_ of adjusted *p*-values. All points on or above the dashed line have a *p*-value of ≤ 0.05 and all points within the light grey shaded region have a *p*-value of ≤ 0.05 and log_2_ fold change ≥ 2. Metabolite classes are indicated by point shape and color. n = 2,198 metabolites.

Supplemental Figure 4. Volcano plot of differential semen metabolite abundance between common bottlenose dolphins (*Tursiops truncatus*) Male 2 and 4. The x-axis represents the log_2_ fold change in metabolite abundance and the y-axis represents the -log_10_ of adjusted *p*-values. All points on or above the dashed line have a *p*-value of ≤ 0.05 and all points within the light grey shaded region have a *p*-value of ≤ 0.05 and log_2_ fold change ≥ 2. Metabolite classes are indicated by point shape and color. n = 2,198 metabolites.
